# Mitochondrial metabolism as a potential therapeutic target in myeloid leukaemia

**DOI:** 10.1038/s41375-021-01416-w

**Published:** 2021-09-24

**Authors:** Lucie de Beauchamp, Ekaterini Himonas, G. Vignir Helgason

**Affiliations:** Wolfson Wohl Cancer Research Centre, Institute of Cancer Sciences, University of Glasgow, Glasgow, UK

**Keywords:** Leukaemia, Cancer metabolism, Cancer stem cells

## Abstract

While the understanding of the genomic aberrations that underpin chronic and acute myeloid leukaemia (CML and AML) has allowed the development of therapies for these diseases, limitations remain. These become apparent when looking at the frequency of treatment resistance leading to disease relapse in leukaemia patients. Key questions regarding the fundamental biology of the leukaemic cells, such as their metabolic dependencies, are still unresolved. Even though a majority of leukaemic cells are killed during initial treatment, persistent leukaemic stem cells (LSCs) and therapy-resistant cells are still not eradicated with current treatments, due to various mechanisms that may contribute to therapy resistance, including cellular metabolic adaptations. In fact, recent studies have shown that LSCs and treatment-resistant cells are dependent on mitochondrial metabolism, hence rendering them sensitive to inhibition of mitochondrial oxidative phosphorylation (OXPHOS). As a result, rewired energy metabolism in leukaemic cells is now considered an attractive therapeutic target and the significance of this process is increasingly being recognised in various haematological malignancies. Therefore, identifying and targeting aberrant metabolism in drug-resistant leukaemic cells is an imperative and a relevant strategy for the development of new therapeutic options in leukaemia. In this review, we present a detailed overview of the most recent studies that present experimental evidence on how leukaemic cells can metabolically rewire, more specifically the importance of OXPHOS in LSCs and treatment-resistant cells, and the current drugs available to target this process. We highlight that uncovering specific energy metabolism dependencies will guide the identification of new and more targeted therapeutic strategies for myeloid leukaemia.

## Introduction

Chronic and acute myeloid leukaemia (CML and AML) are clonal haematological malignancies, born out of genomic aberrations appearing in haematopoietic stem and progenitor cells. While CML is characterised by the presence of the Philadelphia chromosome, the *BCR-ABL* fusion oncogene, AML patients can present with a wide variety of genetic mutations and chromosomal rearrangements.

Today, a majority of CML patients benefit from tyrosine kinase inhibitors (TKIs) which target the constitutively active BCR-ABL protein, which have brought the 5-year survival rate of CML patients above 80% [1]. However, if CML transitions from chronic phase (CP-CML) to blast phase (BP-CML), survival rates drop to a dismal 20% [2]. Regarding AML, the first line of treatment has not radically changed in the past fifty years [3]. Termed ‘7 + 3’ induction therapy, it consists of a nucleoside analogue (such as cytarabine) and an anthracycline (usually daunorubicin) combination treatment. This is often followed by consolidation rounds of the same treatment [4] and, when patients meet eligibility criteria, haematopoietic stem cell (HSC) transplantation [5]. Regrettably, the 5-year survival rate of AML patients remains stubbornly low, averaging 40% for patients under the age of 60, and only 5–15% for older patients [6].

Since the approval of imatinib (Gleevec) by the Food and Drug Administration (FDA) in 2001 [7], novel CML treatment options have mostly consisted in several generations of TKIs [8, 9]. Several innovative AML treatments have however been developed in the past decade. These include hypomethylating agents such as azacytidine [10], several FLT3 TKIs [11, 12], IDH1 and IDH2 inhibitors [13, 14], BCL-2 inhibitor venetoclax [15] and CD33 and CD47 monoclonal antibody conjugates [16, 17]. A Phase I/II clinical trial evaluating chimeric antigen receptor T (CAR-T) cell therapy targeting CD123 is also currently recruiting (NCT04109482).

Understandably, the astonishing efficacy of BCR-ABL TKIs in CML and the recent approval of a series of novel AML treatments have been the source of great hopes for clinicians and patients alike. Yet, major challenges still stand in the way of improving CML and AML patient outcomes and quality of life. These include disease persistence, originating from the inability of standard treatments to target leukaemic stem cells (LSCs), and the indirect selection of treatment-resistant leukaemic cells.

Originally, AML LSCs were defined as leukaemia-initiating cells capable of engrafting in severely immunodeficient mice [18]. Importantly, LSC transcriptional and epigenetic signatures have since been identified, which are largely mutation-independent and are associated with poor clinical outcomes, highlighting the importance of this cell population in disease persistence [19–23]. In CML, disease relapse following TKI treatment discontinuation is attributed to quiescent LSCs, which do not rely on BCR-ABL signalling for their maintenance [24, 25]. In AML, clones carrying early mutations, also known as pre-LSCs, have been identified as a reservoir for disease relapse [26].

Besides LSC persistence, the indirect selection of treatment-resistant cells during therapy is the most complex issue faced in myeloid leukaemia. While LSCs can be considered drug-resistant cells, with the example of quiescent, BCR-ABL kinase-independent CML LSCs [24, 25], not all resistant cells are LSCs. This is exemplified by cells carrying point mutations on the BCR-ABL or FLT3-TKD genes, which are de facto resistant to TKI treatment [27, 28], and by resistance to BCL-2 inhibitor venetoclax [29] or to standard chemotherapy [30, 31] in AML. Indeed, all these resistance mechanisms have been observed in both bulk and LSC populations.

Critically, while the development of mutation-specific treatments has improved the outcome of a large number of CML and AML patients, these targeted therapies have not yielded the widespread clinical success many had hoped for [32, 33]. In AML specifically, this can be attributed to the rapidly evolving nature of the disease, the limited use of deep sequencing techniques for disease monitoring, which is necessary for identifying newly emerging clones, and the sheer lack of targeted therapeutic options for many genetic mutations. As described previously, LSCs and treatment-resistant cells however remain one of the leading causes for our persistent failure to eradicate myeloid leukaemia.

Thus, the study of mutation-independent traits unique to malignant cells appears to be a particularly relevant strategy in the search for new therapeutic options in CML and AML. This is exemplified by the success of anti-apoptotic drug venetoclax [34, 35], which has shown efficacy in a variety of AML subtypes as part of combination treatment regimens. Over 60 trials studying venetoclax in AML alone or in combination are now ongoing. One such specificity of malignant cells, which is now considered a hallmark of cancer, is a rewired cellular metabolism [36], and the importance of this process has been increasingly recognised in haematological malignancies. While the metabolic rewiring associated with leukaemogenesis is extensive, oxidative phosphorylation (OXPHOS) now appears to play a significant role in LSC maintenance and treatment resistance in myeloid leukaemia. The present review will therefore focus on the importance of OXPHOS in LSCs and treatment-resistant cells, and on the drugs currently available to target this process.

## Major metabolic pathways

### Energy metabolism and normal haematopoiesis

**Fig. 1:**
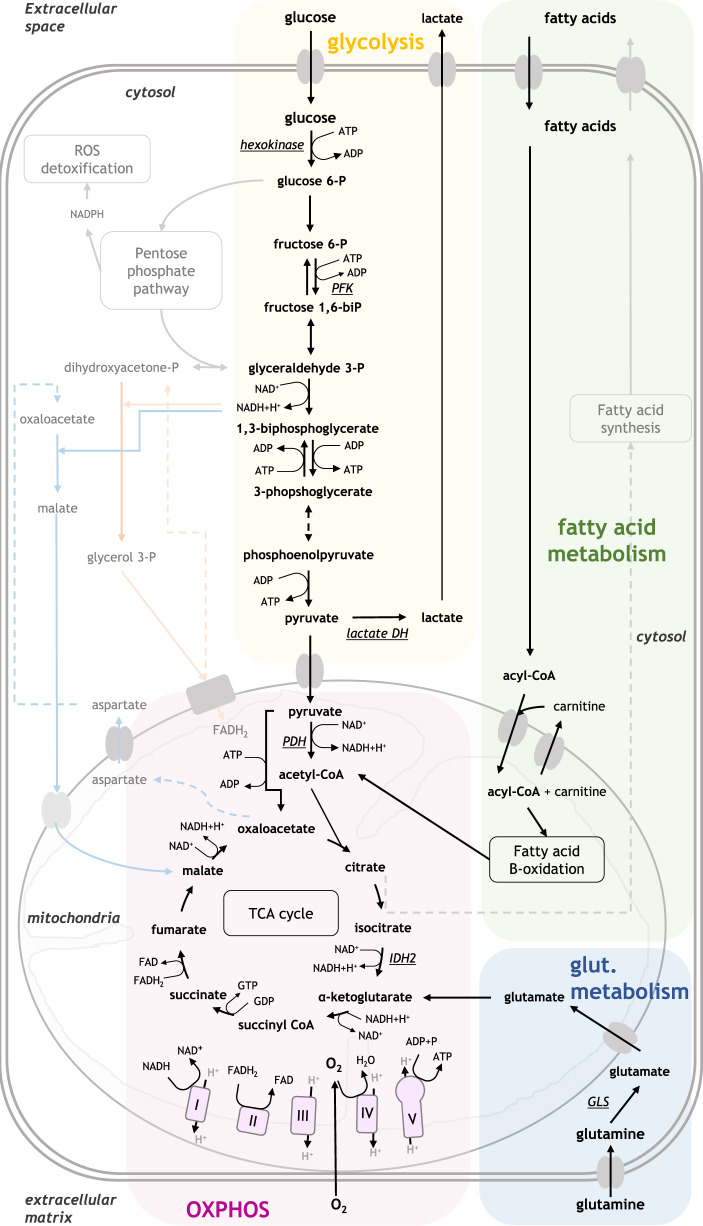
Cellular energy metabolism: Linkage of OXPHOS to catabolic pathways for glucose, fatty acids, and glutamine. Cells use OXPHOS to generate ATP, through the interlinkage of TCA cycle and electron transport chain (ETC). TCA cycle supplies NADH and FADH_2_ to the ETC. Electrons are donated by NADH to complex I and by FADH_2_ to complex II, then transferred to coenzyme Q, to complex III, to cytochrome c and finally to complex IV. This electron transport allows a series of oxidation and reduction reactions within complexes I, II and IV, which in turns allows these complexes to transfer hydrogen protons from inside the mitochondria to the mitochondrial intermembrane space. The accumulation of protons in this space creates a difference in the charge between the inner mitochondria and its intermembrane space. This mitochondrial potential allows protons to flow back into the mitochondria through complex V, providing the energy to bond an inorganic phosphate to a molecule of ADP, producing ATP. Glycolysis is the metabolic process in which glucose is converted into pyruvate, which can then convert pyruvate into acetyl-CoA used in TCA cycle, hence in OXPHOS. One molecule of fructose-6P yields two glyceraldehyde 3-P, thus, one molecule of glucose can yield two molecules of pyruvate. Fatty acid metabolism can also supply the TCA cycle with acetyl-CoA through the fatty acid β-oxidation. Glutamine metabolism is the process in which glutaminase (GLS) converts glutamine to glutamate, which can then be passed into the mitochondrion through the glutamate shuttle and can be converted into α-ketoglutarate, further supplying TCA cycle and enhance OXPHOS activity. (GLS: glutaminase; IDH2: isocitrate dehydrogenase: Lactate DH: lactate dehydrogenase; PFK: phosphofructokinase).

Energy metabolism can be broadly defined as the collection of pathways with at least one product destined for energy production, in the form of ATP (Fig. 1). Approximately 80% of all cellular ATP in our cells is produced by the combination of two processes: the tricarboxylic acid (TCA) cycle, or Krebs cycle, and OXPHOS, powered by the electron transport chain (ETC) [37]. For the ETC to function, a steady supply of NADH and FADH_2_ is required to donate electrons to oxygen, and these cofactors are made available by TCA cycle activity. In turn, for the TCA cycle to function, it needs a steady supply of oxaloacetate and acetyl coenzyme A (acetyl-CoA) [38]. Importantly, cells can synthesise acetyl-CoA from three main sources: carbohydrates, lipids, and proteins, following different catabolic pathways. In case of an imbalance between acetyl-CoA and oxaloacetate, TCA cycle intermediates can be synthesised via parallel pathways, a process known as anaplerosis [38]. Therefore, the linkage of OXPHOS with other catabolic pathways, including glycolysis, fatty acid metabolism and glutamine metabolism is crucial for the cellular energy metabolism in which cells generate energy and survive (Fig. 1).

While essential for virtually all cells in the human body, TCA cycle and OXPHOS activity are linked to and support different processes in each cell type. In haematopoiesis for example, HSCs tend to be glycolytic with low levels of OXPHOS when they are in their dormant state and go through a metabolic switch towards a more oxidative phenotype when they differentiate [39, 40]. To survive in the hypoxic BM niche environment and maintain their integrity, HSCs rely on the anaerobic glycolysis pathway to meet their energy demands [41]. At the transcriptional level, the expression of transcription factor MEIS1 promotes the expression of HIF-1α [41]. Supporting this, the maintenance of HSC quiescence is dependent on PDK-mediated glycolysis [42]. Of note, cell-state-specific metabolic dependencies do not only exist in leukaemogenesis, but also in normal haematopoiesis, as modulations in glucose metabolism sensitise stem cells and progenitor cells differently [43]. ΗSCs also have low OXPHOS levels, due in part to the expression of steroid receptor coactivator 3 [44]. It has been hypothesised that low OXPHOS levels protect HSCs from damaging reactive oxygen species (ROS) production. Indeed, ROS are a known culprit for mitochondrial DNA mutations, and these mutations can inhibit HSC differentiation [45]. To add to this, low ROS cells are enriched for primitive HSCs [46], and HSCs can transfer excess ROS to neighbouring stromal cells [47]. However, challenging the notion that HSCs are glycolytic and have low OXPHOS and ROS levels, cellular mitochondrial content has been shown to be high in quiescent HSCs [48, 49] and HSC quiescence maintained by high levels of large lysosomes rather than high glycolysis [50] emphasising the importance of using accurate assessments of dynamic biological processes in rare cell populations [51].

## Myeloid LSC metabolism

### OXPHOS and mitochondrial biogenesis in CML LSCs

Considering the importance of energy metabolism regulation in HSCs, the study of energy metabolism of LSCs appears particularly relevant. Strikingly, one of the most remarkable observations emerging from the study of myeloid LSCs’ metabolism is the importance of OXPHOS for these cells. Kuntz et al. have for example showed that CD34^+^ CML LSCs have increased TCA cycle flux and mitochondrial respiration compared to their CD34^−^ counterparts [52]. The authors also found that the more primitive CD34^+^ CD38^−^ LSCs relied on OXPHOS significantly more than non-leukaemic HSCs, in keeping with HSCs having a glycolytic metabolic profile [41]. Of particular interest is that integrin-linked kinase, a protein located in focal adhesions which mainly functions as a scaffold through cell-matrix interactions, may regulate mitochondrial metabolism and be a potential therapeutic target for quiescent CML LSCs that are nonresponsive to TKI treatment [53].

In a single-cell RNA sequencing study, Giustacchini et al. elegantly showed that within the same patient-derived CML sample, BCR-ABL+ LSCs overexpressed genes were associated with OXPHOS in comparison to non-malignant HSCs [23]. This is in line with global mRNA microarray analysis using HSCs and progenitor cell populations from CP-CML, BP-CML and non-leukaemic donors, which previously revealed that CP-CML LSCs exhibit an oxidative phenotype with highly expressed mitochondrial respiratory chain (MRC) genes compared to non-leukaemic HSCs [54]. Another outstanding transcriptome profiling revealed that microRNAs (miRNAs) are deregulated in TKI-nonresponsive CML LSCs compared to TKI-responsive CML LSCs, with miR-185 being the most deregulated one. Interestingly its restored function impaired the drug-resistant CML LSCs [55]. To add to this, the authors revealed *PAK6* to be the target gene of miR-185 that enhances the drug resistance in TKI-nonresponsive LSCs and along with the identification of upregulated expression of OXPHOS and ROS in TKI-nonresponsive LSCs, suggests that miRNAs and their target genes can play critical roles in the regulation of mitochondrial metabolism in CML, including in drug-resistant LSCs [55]. In addition, a recent study by Abraham and colleagues found that upregulated OXPHOS in CML LSCs was partly regulated by the NAD-dependent deacetylase sirtuin-1 (SIRT1) [56]. The authors demonstrated that SIRT1 expression activates transcription factor PGC-1α, directly or in an AMPK-dependent manner. PGC-1α then promotes the expression of mitochondrially encoded genes involved in mitochondrial respiration, and increases ETC activity [56]. Direct PGC-1α inhibition however did not fully inhibit CML pathogenesis in vivo, as opposed to SIRT1 inhibition. A previous study by the same group had identified the importance of SIRT1 in CML LSCs through its ability to inhibit p53, highlighting the multiple roles played by this enzyme [57]. Of note, upregulation of OXPHOS in a SIRT1/PGC-1α manner has also been observed in metastatic breast cancer cells which further accentuates their role in OXPHOS and mitochondrial biogenesis, not only in myeloid leukaemia [58].

Lastly, a study which reported mitochondrial transfer from stromal to CML cells revealed that only a small percentage of CML cells acquired mitochondria through this mechanism [59]. Whether this small mitochondria-receiving population is enriched in CML LSCs is an intriguing question and warrants further investigation.

### OXPHOS and regulation of mitochondrial dynamics in AML LSCs

In AML, it has been shown that leukaemia-initiating cells are sensitive to AMPK inhibition and glucose transport reduction [60]. However, many studies have also revealed the importance of OXPHOS [61, 62] and mitochondrial transfer [63, 64] in AML LSCs. Notably, Lagadinou et al. have shown that although LSCs are metabolically less active than the bulk cell population, they are unable to mobilise glycolysis and therefore rely on OXPHOS [65]. In addition, a recent proteomic-based comparison of AML LSCs with HSCs and AML blasts has revealed that several components of ETC complexes I and V were consistently more abundant in LSCs [66]. Incidentally, the importance of OXPHOS in primitive cancer cells has also been observed in transformed mesenchymal stem cells (MSCs) [67], brain tumours [68] and breast cancer [69], highlighting that this metabolic pathway is critical for cancer stem cells (CSCs) in a variety of malignancies [70].

The tight regulation of mitochondrial dynamics is also critical for AML LSCs. Indeed, these cells display a unique mitochondrial morphology compared to bulk AML cells [71]. Furthermore, mitochondrial fission mediated by the mitochondrial fission 1 protein (FIS1) and downstream autophagy is necessary for AML LSCs survival and leukaemia-initiating capacity [71]. To add to this, the epigenetic regulation of mitochondrial fission is also essential to prostate CSCs, highlighting that this process can be critical to CSC maintenance throughout all cancer types [72]. Intriguingly, several studies have shown that unlike HSCs, AML LSCs have a lower mitochondrial content than the bulk AML population [61, 71]. This could be mediated by FIS1 clearance of dysfunctional mitochondria. Importantly, FIS1 expression is associated with poor prognosis in AML patients, and could constitute an indirect marker of LSC signature [73].

Interestingly, entry of pyruvate into the mitochondria by transporter MTCH2 has been linked to AML LSC survival and differentiation [74]. Indeed, Khan et al. have shown that inhibition of MTCH2 not only leads to an accumulation of pyruvate in the cytoplasm, but also to an increase of pyruvate dehydrogenase (PDH) in the nucleus. This increase then promotes histone acetylation, and results in LSC differentiation and reduced survival [74]. Of note, MITCH2 is also involved in embryonic stem cell and HSC differentiation [75, 76]. Another pathway linked to mitochondrial transport is the folding of imported proteins in the mitochondrial intermembrane space. Recently, Singh and colleagues showed that some genes encoding for intermediates of this pathway were upregulated in AML LSCs [77]. Investigating this further, they revealed that inhibition of a copper chaperone involved in this pathway, COX17, increased mitochondrial copper level, decreased S-adenosylmethionine levels and decreased DNA methylation. This in turn reduced LSC viability. However, COX17 inhibition did not affect mitochondrial respiration in LSCs [77]. Taken together, these studies indicate that mitochondrial dynamics can influence epigenetic regulation and survival of AML LSCs, in a respiration-independent manner.

### ROS in myeloid LSCs

In the study by Lagadinou et al. on AML LSCs, the authors found that AML cells with low levels of ROS (whether mitochondrial or not) were enriched for LSCs, regardless of the sample’s mutational background [65]. In keeping with these results, Herault et al. have shown that LSCs have increased expression of the ROS-scavenging enzyme glutathione peroxidase 3 [78]. In another study, the colony-forming and leukaemia-initiating capacity of primary AML cells was inhibited when cells were supplied with high concentrations of palmitate [62]. The study’s authors showed that this was due to an overproduction of OXPHOS-related ROS, which was particularly toxic to AML LSCs, in keeping with AML LSCs residing in a ROS low cells population [65]. In addition, this could explain why FIS1-mediated clearance of dysfunctional, potentially ROS-producing mitochondria is so important to AML LSCs [71].

A report has also shown the importance of the ROS-producing enzyme NOX2 in some AML subsets, both in bulk cells and LSCs [79]. This could be linked to the NOX2-dependent mitochondrial transfer from BM stromal cells to AML cells, including LSCs, shown to be critical for leukaemogenesis [63, 64]. This indicates that the site of production of ROS (mitochondrial ETC or cytosolic enzymes such as NOX2) might influence their role in AML LSC maintenance.

In CML, one report has shown that stem and progenitor cells have higher levels of ROS than bulk cells [80]. The authors found that this is due to the activation of GTPase enzyme Rac2, which promotes ROS production by ETC complex III. In addition, the overexpression of subunit Rac1 of the ROS-producing enzyme NOX2 is linked to CML blast survival, especially in BP-CML [81]. Moreover, a study demonstrated that curcumin, which has anti-tumorigenic effects, specifically inhibits tumour growth in CML, by inhibiting metabolic enzymes involved in ROS pathways, and hence by increasing ROS levels in CML cells [82].

Whether mitochondrial and cytosolic ROS found in CML and AML LSCs are a mere by-product of OXPHOS, a consequence of deregulated oncogenic activation or are implicated in cellular signalling remains largely unknown and calls for further investigation.

### TCA cycle substrates and myeloid LSCs

LSCs also rely on different fuel sources than the bulk leukaemic cell population. Indeed, in a study by Shafat et al., primary AML cells co-cultured with adipocytes prior to xenotransplantation were able to engraft 4 to 6 weeks earlier than cells that had not been co-cultured [83]. This could constitute proof that adipocytes, and potentially the fatty acids they secrete, help maintain the LSC compartment. Supporting this hypothesis, Ye and colleagues have shown that a subpopulation of AML and BP-CML LSCs preferentially locates in extramedullary adipose niches, and overexpresses the fatty acid transporter CD36 [84]. Both studies found increased β-oxidation and TCA cycle activity in LSCs, highlighting that these cells are likely using adipocyte-derived fatty acids to fuel the TCA cycle and the ETC. Importantly, one study has shown that fatty acid oxidation (FAO) inhibition supresses LSC quiescence in primary AML samples in vitro, and sensitises them to apoptosis in vivo [85]. In addition, intracellular levels of phospholipids, regulated in part by the mitochondrial transacylase tafazzin, have been shown to affect AML LSCs stemness while sparing that of HSCs [86]. This mechanism however seemed OXPHOS-independent. In CML, lipoxygenases ALOX5 and ALOX15 have been found to be upregulated in LSCs and to be more important to their survival than that of HSCs [87, 88]. However, to our knowledge, no study has clearly established that the upregulation of these enzymes results in an upregulation of FAO to fuel the TCA cycle and OXPHOS in CML LSCs. Metabolomic studies on this topic are warranted.

Lastly, a study by Jones et al. found that amino acid catabolism is a source of TCA cycle intermediates and a fuel for mitochondrial respiration in AML LSCs, but not in AML blasts nor HSCs [89]. Overall, these studies highlight the extensive metabolic rewiring undergone by LSCs, enabling them to use a wider source of TCA cycle substrates than differentiated blasts or healthy HSCs.

### Conclusion and perspectives on myeloid LSCs metabolic rewiring

Many cancer scientists studying oncometabolism continue to develop Warburg’s aerobic glycolysis model. Vander Heinden et al. for example hypothesise that the increase in glycolysis observed in cancer cells is not used for ATP generation but rather, to provide rapidly dividing cells with all the intermediate metabolites necessary for the production of biomass, particularly nucleotides [90]. While mostly rooted in studies in solid cancers, this theory could be applicable to most myeloid leukaemia cells, particularly the rapidly expanding AML blasts [91, 92]. However, there is now accumulating evidence that AML cells and particularly AML LSCs, as well as CML LSCs, are dependent on OXPHOS and not on glycolysis [52, 65]. As AML LSCs are relatively quiescent and metabolically inactive, this could explain why they might not require such high levels of metabolic intermediates and therefore, why they do not rely on glycolysis [23, 65, 93]. The fact that HSCs, which are also quiescent [94, 95], have been known to rely on glycolysis [41] can question this hypothesis, although whether HSCs share exactly the same BM niche interactions as LSCs, which can affect metabolic requirements, remains to be fully elucidated.

Overall, malignant transformation in both CML and AML is accompanied by a profound metabolic rewiring, resulting in an increased reliance on OXPHOS in leukaemic cells, especially LSCs. Importantly, an increasingly large body of evidence shows that OXPHOS is not only implicated in leukaemogenesis, but also in treatment resistance.

## Treatment-resistant cell metabolism

The notion that leukaemic cells can adapt their metabolism following drug treatment is not new. This is exemplified by the work of Burke and colleagues on the metabolism of patient-derived leukaemic cells treated with oncology drugs, published in the 1950s [96]. However, our current understanding of the metabolic rewiring accompanying leukaemogenesis sheds new light on this idea. OXPHOS-related signatures have for example been associated with poor prognosis, such as high FIS1 expression levels being associated with poor prognosis in AML [73], highlighting the relevance of OXPHOS in treatment resistance.

### OXPHOS in treatment-resistant leukaemic cells

Multiple studies have demonstrated the significant connection between treatment resistance and the fact that leukaemic cells metabolically adapt and survive treatment. More particularly, Roca-Portoles and colleagues showed that venetoclax treatment, which selectively inhibits BCL-2 causing cell apoptosis, decreases mitochondrial respiration, induces inhibition of the TCA cycle, and activates reductive carboxylation in BCL-2 deficient cells [97]. This suggests that venetoclax treated cancer cells may undergo metabolic reprogramming in a manner independent of BCL-2 inhibition and in the absence of cell death. Further studies using various BCL-2 inhibitors did not indicate any metabolic reprogramming like that of venetoclax and highlighted that such metabolic adaptations were dependent on the integrated stress response and the ATF4 transcription factor [97]. Additionally, a recent study reported that AXL, a receptor tyrosine kinase, is highly activated in AML primitive cells and illustrated that treatment with AXL inhibitor alone or in combination with venetoclax seems to eradicate AML primitive cells. In addition, single-cell RNA sequencing analysis revealed that this eradication may be caused by the perturbation of OXPHOS in AML primitive cells [98].

### Potential metabolic influences from the BM microenvironment

LSCs interact with HSCs and other cells present in the BM microenvironment using various mechanisms, involving cytokines, chemokines and adhesion molecules, influencing their cellular metabolic functions [99]. Even though the role of the BM niche in CML/AML metabolism compared to normal haematopoiesis is not yet fully understood, it is widely accepted that BM interactions with AML cells play a significant role in the maintenance and the progression of treatment-resistant AML. Indeed, Kumar and colleagues demonstrated that AML blasts remodel the BM niche and promote leukaemia growth and at the same time suppress normal haematopoiesis through exosome secretion [100]. Interestingly, another study by van Gastel et al. showed that persisting residual AML cells undergo short-term metabolic adaptations as with the help of bone marrow stromal cells (BMSCs) they use glutamine metabolism to survive [101]. More specifically, after chemotherapy, BMSCs supply AML cells with aspartate, resulting in pyrimidine and glutathione generation, hence enabling the survival of residual AML cells. Both glutamine and pyrimidine metabolism pathways were targeted in patient-derived xenografts and leukaemia mouse models and results showed that survival rates were improved in a time-specific manner [101]. In another study, Forte and colleagues demonstrated that in an in vivo AML model, BMSCs support LSCs through OXPHOS, TCA cycle and glutathione-dependent antioxidant defence, promoting leukaemogenesis and chemoresistance [102]. Moreover, Jones and colleagues demonstrated that during AML relapse, AML LSCs are highly dependent on nicotinamide metabolism, which promotes amino acid and FAO metabolism pathways, leading to OXPHOS [103]. As a result, LSCs from relapsed AML patients are found to be resistant to venetoclax and azacytidine combination treatment. Therefore, it is critical to understand how leukaemic cells depend on OXPHOS as a metabolic reprogramming pathway, as this could provide opportunities to metabolically disrupt persistent leukaemic cells and overcome chemoresistance.

Importantly, studies have found that the apparent metabolic signatures of LSCs are unaffected by treatment. For example, the high levels of SIRT1 expression, which regulates mitochondrial biogenesis and OXPHOS in CML LSCs, remain unchanged in these cells upon TKI treatment [56]. One report by Jones et al. found that treatment-resistant AML LSCs were able to maintain OXPHOS levels necessary to their survivals throughout treatment, by using FAO instead of amino acids as a TCA cycle fuel source [89]. Treatment-sensitive LSCs did not present the same substrate flexibility [89]. In addition, FLT3 TKI-resistant AML LSCs use glutaminolysis to fuel the TCA cycle, highlighting that treatment-resistant cells can use a variety of substrates to supply OXPHOS [104]. Lastly, AML and BP-CML LSCs residing in extramedullary adipose niches and overexpressing the fatty acid transporter CD36 are significantly more chemo-resistant than their BM niche counterparts [84]. This shows that the metabolic interplay between LSCs and the BM niche is also implicated in treatment resistance. However, OXPHOS-related treatment resistance is not limited to LSCs, going against the view that treatment resistance in leukaemia is solely due to LSC persistence. For example, mitochondrial transfer between BM stromal cells and AML bulk cells confer the receiving cells with increased OXPHOS capacity and increased resistance to treatment [63]. In CML, while only a small proportion of leukaemic cells have been shown to receive mitochondria from stromal cells, the transfer of vesicles via nanotubes protects CML cells from imatinib-induced apoptosis [59]. Whether mitochondrial transfer protects the recipient cells from treatment-induced apoptosis is, therefore, a legitimate question.

In an elegant study using a large number of primary AML samples and xenotransplant experiments, Farge and colleagues have shown that cytarabine-resistant cells are not enriched for quiescent LSCs, but instead for cells with high OXPHOS [30]. Importantly, this held true both at the transcriptional and phenotypical levels [30]. In keeping with other reports [84], the authors also found that treatment-resistant AML cells overexpressed CD36 and other fatty acid transporters. Interestingly, this same study showed that while treatment-naïve cells had heterogenous OXPHOS profiles, the majority of cells had high OXPHOS after treatment [30]. These results however did not allow determining whether cytarabine treatment had selected for OXPHOS-high cells or led to an increase in OXPHOS in all cells. In a more recent study from the same group, the authors not only confirmed the increase in OXPHOS in chemo-resistant AML cells but also revealed the key role of extracellular ATPase CD39 in the increase in mitochondrial biogenesis and respiration [105]. Interestingly, the authors also highlighted that this CD39-mediated metabolic resistance mechanism appeared to be cell-intrinsic, meaning that cytarabine treatment likely selected cells already utilising this pathway rather than activating it in all treated cells. In addition, Baccelli and colleagues recently showed that chemo-resistant AML cells are particularly sensitive to mubritinib-mediated inhibition of ETC complex I [106]. Lastly, OXPHOS-associated treatment resistance is also observed in diffuse large B cell lymphoma, highlighting that this resistance mechanism exists throughout haematological malignancies [107, 108].

### Energy metabolism and apoptosis resistance

The importance of OXPHOS is not only observed in chemotherapy-resistant malignant cells. In chronic lymphocytic leukaemia for example, resistance to BCL-2 inhibitor venetoclax is associated with an increase in OXPHOS transcriptional signature, in addition to an overexpression of MCL-1 [109]. In multiple myeloma, increased activity of ETC complexes I and II predicts poor response to venetoclax treatment, and inhibition of either complexes sensitises cells to BCL-2 inhibition [110]. In addition, inhibition of mitochondrial translation sensitises resistant AML cells to venetoclax [111]. While the exact mechanism remains only partially understood, a link between OXPHOS levels and BCL-2 inhibition response has now been clearly established in several cancer models [109, 110, 112].

The normal intrinsic apoptosis pathway is as follows: when a pro-apoptotic signal arises in a cell, sequestrated pro-apoptotic BH3-only proteins (including BIM, BID, BAD and PUMA) are released from anti-apoptotic proteins of the BCL-2 family (including BCL-2, BCL-xL and MCL-1), triggering BAX and BAK activation, mitochondrial membrane permeabilization and cytochrome c release [113]. The resistance to apoptosis observed in virtually all cancers often involves a deregulation of this machinery [114]. In AML for example, chemotherapy resistance is associated with high levels of BCL-2 expression [115], which is also observed in ROS low cells enriched for AML LSCs [65]. However, Letai and colleagues have shown that resistance to BH3 mimetic treatment is not only dependent on high expression levels of anti-apoptotic proteins, but also on the low number of BH3-only proteins these anti-apoptotic proteins are sequestrating [116–118]. This concept, called BH3 priming, is elegantly summarised in this review by Konopleva and Letai [119]. Critically, while non-malignant cells also express proteins of the BCL-2 family, these appear to have less BH3 priming than cancer cells, explaining why BH3 mimetics are selectively more toxic to cancer cells [117].

Yet, the underlying mechanism linking OXPHOS and apoptosis evasion remains obscure. It has previously been shown that BAD, a BH3-only protein, is part of a glucose-sensing complex that includes the glycolysis enzyme glucokinase [120]. The study’s authors showed that in hepatocytes, physiological glucose levels ensured glucokinase activity and phosphorylated BAD, inactivating it. Glucose deprivation led to BAD dephosphorylation and activation, triggering glucose deprivation-dependent apoptosis [120]. Other studies have linked aerobic glycolysis and apoptosis resistance [121], including in BCR-ABL+ cells treated with imatinib [122, 123]. Interestingly, it has also been shown that malignant AKT signalling could only prevent apoptosis in the presence of high levels of energetic pathways’ metabolites [124]. Specifically, BH3-only protein PUMA could only be inhibited by AKT in the presence of glucose or other TCA cycle intermediates [124]. The authors speculated that both PUMA expression and stabilisation could be regulated in a p53-dependent or independent manner, but that both mechanisms were linked to cellular energetic status [124]. In addition, Bajpai and colleagues have shown that glutaminolysis inhibition sensitises multiple myeloma cells to venetoclax, by increasing BIM expression and its sequestration by BCL-2 [125]. The authors suggest that this is due to a decrease in α-ketoglutarate levels, and further investigation of this mechanism is warranted [125]. Interestingly, glutaminolysis inhibition also synergises with venetoclax in AML [126]. Of note, culture of primary AML cells with FLT3 ligand protects them from serum starvation-induced apoptosis, by preventing BAX upregulation [127]. This could be due to increased BH3 sequestration by BCL-2, preventing BAX activation. In addition, Gallipoli and colleagues have shown that when treated with FLT3 TKIs, FLT-TKD-mutated AML cells increase their glutaminolysis flux, and co-inhibition of FLT3 signalling and glutaminolysis increased cell death [104]. Whether this dual inhibition “overwhelms” BCL-2 proteins with BH3-only proteins, rendering them unable to sequestrate all BH3 protein signalling and prevent apoptosis, is an intriguing question. Therefore, to override the need for external pro-survival cytokine signalling, or to silence the intrinsic pro-apoptotic signals sent during DNA breakage induced by chemotherapy, cancer cells could rely on the increased sequestration of BH3-only proteins by anti-apoptotic proteins. As a low energetic status can be an additional trigger for BH3-only protein expression or activation, cancer cells might need to upregulate their energy metabolism flux to avoid yet another pro-apoptotic trigger. While further research is needed to uncover the energy-sensing mechanisms that inform BCL-2 proteins priming, this could represent an exciting new target in overcoming apoptosis resistance in myeloid leukaemia.

## Why do LSCs and treatment-resistant cells rely on OXPHOS?

The fact that CML and AML LSCs rely on OXPHOS regardless of their genetic background is intriguing. While one can understand how tyrosine kinase mutations such as BCR-ABL and FLT3-ITD could activate similar metabolic pathways, it is unclear how the many other mutations found in AML can all lead to a relatively unified metabolic profile. As mutation-independent LSC signatures have been identified at the transcriptional and epigenetic levels, it could be hypothesised that these signatures are echoed at the metabolic level. As these LSC signatures are strong prognosis markers, it would be particularly relevant to investigate their metabolic repercussions. To our knowledge, no such study has yet been undertaken.

While an increase in OXPHOS could help myeloid leukaemic cells evade apoptosis, as discussed above, it might be implicated in other resistance mechanisms. For example, Birsoy and colleagues demonstrated that ETC activity which directly affects mitochondrial metabolism, and hence OXPHOS, might be critical to cells not only for ATP production, but for aspartate synthesis as well, as in the presence of aspartate, ETC-defective cells are able to proliferate [102, 128]. It is yet unclear how this exactly relates to treatment resistance; however, it highlights that even when ETC is inhibited, leukaemic cells can still identify ways to resist targeted inhibition and proliferate [128]. In addition, another study that aimed to understand the link of BCL-2 with mitochondrial respiration with relation to COX in tumour cells, demonstrated that when BCL-2 is overexpressed this leads to relatively high level of COX activity, oxygen consumption rate, and mitochondrial respiration, further indicating the reliance of tumour cells on OXPHOS [129]. Interestingly, a recent large-scale sequencing study characterised the mitochondrial genomes in 38 types of cancer, illustrating the prevalence of mutations in mitochondrial DNA (mtDNA) in cancer [130]. The frequency of mtDNA mutations in leukaemia observed in this study showed the increased role of mtDNA mutations in leukaemic progression.

Furthermore, the important ATP yield of OXPHOS could be used to fuel ATP-dependent drug efflux pumps, in particular the multidrug resistance proteins P-glycoprotein (PGP) and the multidrug resistance protein 1 (MDR1) [131]. This would be in keeping with PGP expression being linked to treatment resistance in both CML [132] and AML [133]. Mixed results obtained with specific PGP inhibitors in AML have been attributed to compensatory mechanisms with other drug efflux pumps such as MDR1 [134]. As drug efflux pumps expression is found throughout cancers [131], this could explain why treatment resistance is associated with an increase in mitochondrial metabolism and OXPHOS in such a wide variety of pathologies, including myeloid leukaemia [30, 52], lymphoid malignancies [108, 109], breast [69, 135], prostate [72] and lung cancer [136]. To our knowledge, no study has however established a clear link between OXPHOS-derived ATP production, drug efflux pumps and treatment resistance in leukaemia, and this calls for further investigation.

In conclusion, while normal HSCs rely predominantly on glycolysis [41], AML and CML LSCs, and treatment-resistant cells, appear OXPHOS-dependent [30, 52] and unable to mobilise glycolysis [65]. As LSC persistence and therapy resistance remain the greatest challenges in the treatment of myeloid leukaemia, OXPHOS appears to be a clinically relevant target in these malignancies.

## Existing/ongoing clinical developments

**Fig. 2:**
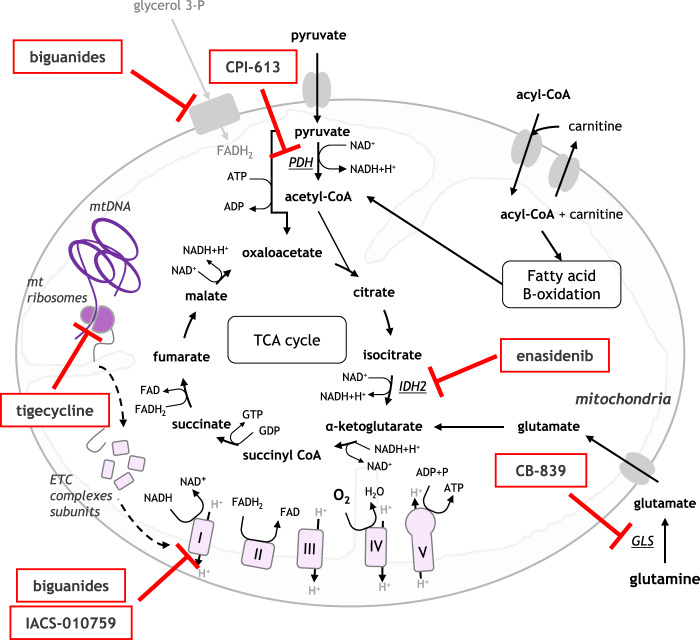
The specific activity of OXPHOS inhibitors tested in the clinic in leukaemia. Representation of mechanism of action of each compound and their clinical trial status (Table 1). (GLS: glutaminase; IDH2: isocitrate dehydrogenase; mtDNA: mitochondrial DNA; mtRibosomes: mitochondrial ribosomes; PDH: pyruvate dehydrogenase).

As the relevance of targeting OXPHOS in several cancers, including myeloid leukaemia, has become apparent, interest has grown for the development of clinically applicable OXPHOS inhibitors. While IDH inhibitors have shown some indirect effects on cellular metabolism [137], their therapeutic success is rather rooted in their hypomethylating effects. BCL-2 inhibitor venetoclax has also been shown to affect mitochondrial respiration [89]. As previously mentioned, Roca-Portoles and colleagues have recently demonstrated that this effect may be BCL-2-independent, and instead mediated by the integrated stress response via transcription factor ATF4 [97]. While this mechanism has yet to be fully confirmed in myeloid leukaemia, it is possible that venetoclax efficacy in these diseases is, at least in part, due to its effect on mitochondrial respiration. In addition to IDH inhibitors and venetoclax, other drugs, old and new, have been studied for their specific activity on OXPHOS in myeloid leukaemia (Fig. 2). The dependency of LSCs and treatment-resistant cells on mitochondrial metabolism led to the testing of these drugs, which based on their specific mechanism of action may directly target the cellular mitochondrial metabolism, which can lead to OXPHOS inhibition (Table 1).

**Table 1:** Overview of existing/ongoing clinical developments.

Compounds	Mechanism of Action	Clinical Trial Results	References
Venetoclax	Selectively inhibits BCL-2 protein	Phase 1b/2 trials of venetoclax plus a hypomethylating agent therapy showed tolerable and promising clinical activity, over 60 trials are currently ongoing	[34, 36]
Biguanides	Inhibits the ETC complex I and the glycerol phosphate shuttle	Metformin trial in ALL relapsed patients showed a protective effect, however only high concentrations found effective in in vitro studies raising safety concerns for lactic acidosis, and phenformin trial in melanoma is currently in progress	[140, 145]
Tigecycline	Inhibits the synthesis of mitochondrial ETC complex subunits encoded by mtDNA	Phase I trial on relapsed/refracted AML patients showed satisfactory safety profile, but unsuccessful clinical benefits of tigecycline	[150]
IACS-010759	Inhibits activity of ETC complex I	Satisfactory efficacy in in vivo AML and glioma models, however reported increased serum lactate levels and currently phase I trials on relapsed/refractory AML in progress	[68, 150]
CPI-613	Deactivates PDH and prevents the entry of acetyl-CoA into TCA cycle	Phase I trials undergone in relapsed/refractory AML patients with CPI-613 alone and in combination with cytarabine with promising results in the combination treatment, phase II trial currently ongoing	[151, 154]
CB-839	Inhibits glutaminolysis by targeting GLS	Pre-clinical data were positive and phase I trial in AML and phase II trial on advance myelodysplastic malignancies are currently ongoing	[155]
Enasidenib	Inhibits the decarboxylation of isocitrate by inhibiting IDH2	Phase I and II trials revealed enasidenib to be tolerable and to induce haematologic responses in relapsed/refractory mutant-IDH2 AML, phase III is ongoing	[13]

*GLS* glutaminase, *IDH2* isocitrate dehydrogenase, *mtDNA* mitochondrial DNA, *PDH* pyruvate dehydrogenase.

### Biguanides

Metformin, a drug routinely used for the treatment of diabetes, has been proposed as a metabolic inhibitor of cancer cells [138]. Indeed in 2005, a study showed that diabetic patients treated with metformin had a reduced risk of developing certain types of cancer [139]. Since then, many hypotheses have been proposed for metformin’s mechanism of action (Fig. 2). The most common explanation is that metformin inhibits complex I of the ETC [140]. While some argue that this leads to a drop in ATP levels and thus AMPK activation [140], others show that this inhibition leads to direct decrease in OXPHOS [141]. In addition, Madiraju et al. have shown that metformin inhibits the glycerol phosphate shuttle [142]. Large variations in metformin concentrations and cancer models could explain seemingly contradictory reports. Nonetheless, Scotland and colleagues have shown that metformin can elicit cell cycle arrest and apoptosis in AML cells in an AMPK-independent manner [143]. Many clinical trials have been undertaken to test metformin in cancer with varying levels of success. In leukaemia, only one trial has, to our knowledge, shown a protective effect of metformin against relapse, in acute lymphoblastic leukaemia (ALL) [144]. In myeloid leukaemia, however, only one trial testing metformin in AML has been recorded. The trial, which aimed to test metformin in combination with cytarabine, commenced in 2013 but was terminated in 2016 because of slow patient recruitment (NCT01849276). The relatively disappointing results of metformin cancer trials could be due to the high concentrations of metformin (from 50 µM to 10 mM) used in the original in vitro studies, which could prove difficult to achieve in patients [140, 141, 143]. In addition, metformin treatment leads to a compensatory increase in glucose uptake and glycolysis in cancer cells [141, 143]. As lactic acidosis is a severe side effect of metformin observed in diabetic patients [145], there are safety concerns associated with the use of metformin in already fragile cancer patients.

Phenformin, a more potent analogue of metformin, has been studied for similar applications in cancer including melanoma [146]. Interestingly, Somlyai and colleagues have highlighted the structural similarity between phenformin and imatinib, proposing that this TKI might actually be targeting metabolic functions of CML cells in addition to BCR-ABL signalling [147]. To date, only one clinical trial studying phenformin for cancer application has been reported, and this trial on melanoma is currently recruiting (NCT03026517). Of note, the FDA withdrew phenformin in the 1970s due to the high risk of severe acidosis [148].

### Tigecycline

Another repurposed drug shown to inhibit OXPHOS is the antibiotic tigecycline. It was first identified for this application by Škrtić et al., in a drug repurposing screen in an LSC-like AML cell line [61]. The authors showed that tigecycline treatment alone or in combination with standard chemotherapy reduced the leukaemic engraftment capacity and tumour burden in xenotransplant assays. The study also revealed that tigecycline’s anti-leukaemic activity was due to its inhibition of mitochondrial protein translation [61]. Indeed, mitochondrial ribosomes bear strong similarities with the bacterial ribosomes that tigecycline inhibits. Tigecycline therefore inhibits the synthesis of mitochondrial ETC complex subunits encoded by mtDNA, and impairs OXPHOS (Fig. 2). Critically, this inhibition was fatal to primitive AML cells but not to normal HSCs [61]. Following this, Kuntz and colleagues showed that tigecycline was also effective in inhibiting OXPHOS in treatment-resistant CML LSCs, both in vitro and in vivo, while sparing HSCs [52]. Unfortunately, a Phase I trial on AML patients fell short of showing the clinical efficacy of tigecycline [149]. While the safety profile of the drug was satisfactory, no clinical benefit of tigecycline was observed in the relapsed and refractory AML patients tested. The authors did not find any evidence of mitochondrial translation inhibition, explaining why no response was obtained in patients. The main hypothesis for the treatment’s failure was the inability to maintain sufficiently high Cmax to allow mitochondrial translation inhibition, as tigecycline’s half-life proved much shorter than previously published [149]. While improved formulation could allow better efficacy in patients, no new clinical trial is currently accruing to test tigecycline for cancer applications.

### IACS-010759

Researchers have also been working towards synthesising purpose-designed OXPHOS inhibitors. This is the case of Molina and colleagues who, out of a collection of over 400 compounds, selected IACS-010759 for its inhibitory activity of ETC complex I (Fig. 2) [68]. This potent compound showed efficacy in the nanomolar range in vitro, and reduced tumour burden in in vivo AML and glioma models [68]. IACS-010759 is currently being tested in two Phase I trials focusing on relapsed/refractory AML (NCT02882321) and various advanced cancers including pancreatic and breast cancers (NCT03291938). While both trials are ongoing, a preliminary report on the second trial states that some patients have shown disease stabilisation and one has shown partial response [150]. While the safety profile appeared overall satisfactory and no severe lactic acidosis has been reported, increased serum lactate levels were observed in most patients. This is in keeping with IACS-010759’s direct inhibition of complex I of the ETC, similar to biguanides’ mechanism of action.

### Other clinical-stage OXPHOS inhibitors

Other OXPHOS inhibitors have reached clinical trial stage. These include CPI-613, a small molecule deactivating PDH and preventing the entry of acetyl-CoA into the TCA cycle, inhibiting OXPHOS (Fig. 2) [151]. CPI-613 has been tested in advanced haematological malignancies in a Phase I trial, where it showed good patient safety but limited efficacy, with most AML patients not reaching treatment response outcome [152]. Following this, CPI-613 was tested in combination with cytarabine in a cohort of relapsed/refractory AML patients in another Phase I study [153]. Based on encouraging results in this study, a Phase II trial was launched in 2019 [154]. In addition, following positive pre-clinical data [155], glutaminolysis inhibitor CB-839 has been tested in combination with azacytidine in a Phase I AML trial (NCT02071927), and a Phase II trial on advance myelodysplastic syndrome is recruiting (NCT03047993).

Pharmacological targeting of OXPHOS metabolism in myeloid leukaemia is therefore an active area of research. Although several molecules have brought proof of concept that this metabolic pathway could be targeted in patients, efficacy has been more difficult to attain in the clinic, and some safety concerns remain. Of note, to our knowledge, no OXPHOS inhibitor has yet been clinically tested for the treatment of CML. Finally, considering the promising results obtained from recent studies regarding targeting mitochondrial metabolism in myeloid leukaemia, it can be concluded that metabolic pathways can be targeted using many different strategies, for instance, by targeting ROS, CD36, or components of the ETC. However, for both CML and AML, the discovery of novel OXPHOS inhibitors used in combination treatments, can potentially overcome the limitations for developing improved and targeted therapeutic strategies.

## Conclusion

While we now have a thorough understanding of the genomic abnormalities underpinning myeloid leukaemia, it has become evident that our knowledge of the biology of leukaemic cells, however, remains incomplete. Energy metabolism rewiring is the perfect example of a process, which is paramount to these cells, while being mostly mutation-independent. Specifically, the importance of OXPHOS in LSCs and treatment-resistant cells in CML and AML has become increasingly apparent. While this has yet to translate into clinically applicable therapeutic strategies, it is our hope that uncovering specific energy metabolism dependencies and/or their links with the cells’ microenvironment will open new research avenues and, ultimately, new treatment options for CML and AML patients.
